# Longitudinal Sequence and Functional Evolution within Glycoprotein E2 in Hepatitis C Virus Genotype 3a Infection

**DOI:** 10.1371/journal.pone.0126397

**Published:** 2015-05-13

**Authors:** Yousef M. O. Alhammad, Sanvir Maharajh, Rebecca Butcher, John-Sebastian Eden, Peter A. White, Pantelis Poumbourios, Heidi E Drummer

**Affiliations:** 1 Centre for Biomedical Research, Burnet Institute, 85 Commercial Rd, Melbourne, 3004, Australia; 2 Department of Microbiology, Monash University, Clayton, Victoria, Australia; 3 Department of Microbiology and Immunology, The University of Melbourne at the Peter Doherty Institute for Infection and Immunity, Melbourne, Victoria, Australia; 4 School of Biological Sciences and Sydney Medical School, Charles Perkins Centre, The University of Sydney, Sydney, New South Wales, Australia; 5 School of Biotechnology and Biomolecular Sciences, University of New South Wales, Sydney, New South Wales, Australia; University of Montreal Hospital Research Center (CRCHUM), CANADA

## Abstract

The E2 glycoprotein of Hepatitis C virus (HCV) is a major target of the neutralizing antibody (NAb) response with the majority of epitopes located within its receptor binding domain (RBD; 384–661). Within E2 are three variable regions located at the N-terminus (HVR1; 384–411), and internally at 460–480 (HVR2) and 570–580 [intergenotypic variable region (igVR)], all of which lie outside a conserved core domain that contains the CD81 binding site, essential for attachment of virions to host cells and a major target of NAbs. In this study, we examined the evolution of the E1 and E2 region in two patients infected with genotype 3a virus. Whereas one patient was able to clear the acute infection, the other developed a chronic infection. Mutations accumulated at multiple positions within the N-terminal HVR1 as well as within the igVR in both patients over time, whereas mutations in HVR2 were observed only in the chronically infected patient. Mutations within or adjacent to the CD81 contact site were observed in both patients but were less frequent and more conservative in the patient that cleared his/her infection. The evolution of CD81 binding function and antigenicity was examined with longitudinal E2 RBD sequences. The ability of the RBD to bind CD81 was completely lost by week 108 in the patient that developed chronic HCV. In the second patient, the ability of the week 36 RBD, just prior to viral clearance, to bind CD81 was reduced ~50% relative to RBD sequences obtained earlier. The binding of a NAb specific to a conserved epitope located within E2 residues 411–428 was significantly reduced by week 108 despite complete conservation of its epitope suggesting that E2 antigenicity is allosterically modulated. The exposure of non-neutralizing antibody epitopes was similarly explored and we observed that the epitope of 3 out of 4 non-NAbs were significantly more exposed in the RBDs representing the late timepoints in the chronic patient. By contrast, the exposure of non-neutralizing epitopes was reduced in the patient that cleared his/her infection and could in part be attributed to sequence changes in the igVR. These studies reveal that during HCV infection, the exposure of the CD81 binding site on E2 becomes increasingly occluded, and the antigenicity of the E2 RBD towards both neutralizing and non-neutralizing antibodies is modulated via allosteric mechanisms.

## Introduction

Hepatitis C virus encodes two membrane anchored envelope proteins, E1 and E2, that are essential for attachment to target cells, viral fusion and entry, and are targets of the neutralizing antibody (NAb) response during infection. The cell surface tetraspanin CD81 has been shown to be an essential receptor for HCV entry into target cells and directly interacts with residues within the E2 receptor binding domain (RBD), residues 384–661. The HCV E2 glycoprotein encodes three variable regions designated as hypervariable region 1 (HVR1), hypervariable region 2 (HVR2) and the intergenotypic variable region (igVR). HVR1 has been characterized as an immunodominant region of E2 that elicits isolate specific NAbs and is therefore under immune selection pressure. Viral clearance has been attributed to limited HVR1 sequence evolution, with fewer HVR1 mutations correlating with antibody mediated clearance of virus [1]. HVR2 and the igVR have been shown to play a role in HCV assembly [2], are important for E1E2 heterodimerization and HCV infectivity [3]. HVR2 and the igVR form surface exposed flexible regions flanked by cysteine residues [4–6]. However, they are not direct targets of the antibody response and the reason for variation within their sequences is unknown. Nevertheless, it has been shown that the variable regions modulate accessibility of the CD81 binding site [2, 3]. Furthermore, cell culture derived viruses (HCVcc) lacking HVR1 are more sensitive to NAbs and are more accessible to soluble CD81 [7].

The majority of broadly neutralizing monoclonal antibodies isolated from chronically infected humans are directed towards regions involved in CD81 binding interactions. Four discontinuous regions form the CD81 binding site within the E2 core domain and overlap with the epitopes of NAbs [5, 8–11]. The recent three-dimensional structure of the E2 core revealed residues involved in CD81 binding to be located within an α-helix (α1) in the so-called front layer of the E2 core domain and the CD81 binding loop [4, 5]. The CD81 binding residues Trp^420^ and His^421^ overlap with NAb epitopes located within the 411–428 region referred to here as epitope-I. Structural information for epitope-I is absent from E2 core structures [4, 5]. However, structural studies of soluble epitope-I peptides cocrystallised with neutralizing monoclonal antibodies reveal that this region can adopt alternate conformations suggesting that the structural flexibility of HVR1 extends into epitope I, consistent with the inability to resolve the structure of this region in the E2core domain [12–14]. The second CD81 binding site component includes residues from within the Gly^436^—Trp^443^ segment which overlaps with a second conserved NAb epitope (residues 429–446), referred to here as epitope-II [4, 5, 9]. A third component of the CD81 binding site includes Trp^527^, Trp^529^, Gly^530^, and Asp^535^ and overlaps with the region 523–549 referred to here as epitope-III [4, 5, 10]. The CD81 binding site and epitope-II are affected by changes in the Y^613^RLWHY segment. This helical segment Y^613^RLWHY is likely to contribute to the conformation of epitope II and/or directly contacts the CD81 binding site. Neutralizing human and murine MAbs have been isolated that overlap with epitopes I, II and III and inhibit E2-CD81 binding [15].

HCV evades the adaptive antibody response through the acquisition of immune escape mutations. The majority of these mutations are within HVR1 suggesting it is immunodominant [16–19]. In addition, glycoprotein E2 is extensively glycosylated and these glycans can mask neutralization epitopes [20, 21]. Five glycans at 417, 423, 448, 532 and 644 reduce sensitivity to neutralization, four of which surround the CD81 binding site [22]. Glycan shifting has also been observed in E2 isolates passaged with NAb to epitope I. A shift in glycosylation from residue N417 to N415 reduces sensitivity to neutralization despite this residue not contributing to the epitope of the MAb [23]. However, why the sequences of HVR2 and the igVR evolve has not been delineated.

Viral evolution in two HCV infected patients from the Australian Trial In Acute Hepatitis C were examined in this study [24]. Two patients infected with HCV genotype 3a were examined and both were treatment naïve. In this study, longitudinal viral E1E2 sequences were analysed to evaluate the evolution within the discontinuous CD81 binding sites, HVR1, HVR2 and the igVR. The E2 RBD glycoproteins were generated from clones representative of the dominant quasispecies from the earliest and latest available time points. To examine how sequence changes that emerged during infection modulated the E2 RBD structure and function, amino acid changes found in the latest time point within the variable regions or the CD81 binding sites were inserted into E2 RBD clones from the earliest time point. The differences in E2 RBD binding to CD81 and the antigenicity of the E2 RBDs were examined. Our results indicate that E2 RBD sequence evolution alters exposure of CD81 binding sites and both NAb and non-NAb epitopes.

## Materials and Methods

### Patient samples

The Australian Trial in Acute Hepatitis C (ATAHC) is a prospective cohort study of the natural history and treatment of recent HCV infections [24]. Participants with either acute or early chronic HCV infection were enrolled. Participants were screened for potential enrolment and those with detectable levels of HCV RNA at screening were recruited and assessed for treatment. All samples were obtained after written informed consent was obtained from each participant. The ATAHC study protocol was approved by St Vincent Hospital, Sydney Human Research Ethics Committee. Details of the two patients examined in this study are provided in S1 Table. Two patients infected with HCV genotype 3a and were treatment naïve were selected. The first patient was untreated and unable to clear the virus and became chronically infected (patient A). The second patient was untreated but able to clear the virus spontaneously with the last RNA positive time point obtained at week 36 (patient B).

### E1E2 cDNA of HCV infected patients

The cDNA encoding the E1 and E2 regions (amino acids 120–861) was amplified as one fragment by RT-PCR from viral RNA extracted from sera of HCV infected patients using degenerate primers (S2 Table) [25, 26]. The E1E2 cDNA of the patient A was amplified from five time points at screening (SC, week 0, early time point), 36 weeks, 60 weeks, 96 weeks, and 108 weeks (late time point) after the SC time point (S1 and S2 Tables). The E1E2 cDNA of the patient B was amplified from three time points; baseline (BL) obtained four weeks after SC (early time point), 8 weeks and 36 weeks (late time point) (S1 and S2 Tables). The cDNA of each timepoint was ligated into pGEM-T Vector Systems (Promega) and at least twenty independent clones of each timepoint from both patients and both sense and antisense strands were sequenced using big dye terminator chemistry. Dominant sequences were used to analyse sequence evolution. Sequences were analysed using CLC bio Genomics Workbench software. HCV mutations present within the E2 RBD region were studied by comparing consensus sequences to that of the early time point. Evidence of positive selection assessed using the Datamonkey web server of the HyPhy package [27]. Two codon-based maximum likelihood methods were used to estimate the ratio of non-synonymous to synonymous substitutions per site (ratio *d*_N_/*d*_S_); Single likelihood Ancestor (SLAC) and Mixed Effects Model of Evolution (MEME). Sites with *p*-values <0.05 were considered as providing significant evidence of positive selection.

#### E2 RBD plasmids

The E2 RBD was generated from cloned E1E2 cDNA representative of the dominant viral quasispecies for early and late time points from both patients. The E2 RBD constructs were synthesized with Expand High Fidelity PCR system according to the manufacturer’s recommendations (Roche Applied Sciences, Indianapolis, IN) using specific primers (S3 Table). The PCR consisted of 30 cycles at 94°C for 60s, 55°C for 60s, and 72°C for 90s and final extension step at 72°C for 5 min. Site directed mutagenesis of specific residues was performed by overlap extension PCR. Changes in the late time point sequences were introduced into the early time point sequences using internal primers (S3 Table). Individual clones containing an insert of the expected size were sequenced in both sense and antisense strands with big dye terminator chemistry.

### Antibodies and cell lines

Anti E2 mouse monoclonal antibodies (MAb) MAb6, MAb13, MAb22, MAb24, MAb25, and MAb26 were generated and characterized in our laboratory (Alhammad, *et al*. submitted). Rabbit anti His antibody was purchased from Rockland Immunochemicals Inc. (Gilbertsville, PA). The goat anti-mouse Alexa Fluor 680 fluorescent antibody was purchased from Invitrogen, Life Technologies. The goat anti-rabbit IRDye-800 antibody was purchased from Rockland Immunochemicals Inc. The rabbit anti-mouse and goat anti-rabbit antibodies conjugated with horse-radish peroxidase (HRP) were purchased from Dako (Glostrup, Denmark). Human embryonic kidney cells (HEK-293T) were grown in Dulbecco’s modified Eagle’s medium (DMEM) (Gibco Life Technologies) supplemented with 10% heat-inactivated foetal bovine serum (FBS) (Gibco Life Technologies), 2mM L-Glutamine (Gibco Life Technologies) and 1M HEPES buffer solution (Gibco Life Technologies) with addition of Gentamicin and Minocycline HCl antibiotics (DMF10).

### Transfection and expression of E2 RBD glycoproteins

Expression of E2 RBD glycoproteins was performed by transfecting HEK-293T cells with E2 RBD plasmids. Transfection mixture was prepared by combining 35μg of E2 RBD DNA with 105μg of Polyethylenimine (PEI) and 4mL of Opti-MEM, vortexed and incubated for 15 min at room temperature (RT). DMF10 media was removed from the HEK-293T cells seeded in T175 cm^2^ flasks and transfection mixture was added onto the cells and incubated with 20mL of Opti-MEM. Tissue culture fluid containing E2 RBD glycoproteins was collected and exchanged every day for five consecutive days. Expressed E2 RBD glycoproteins were concentrated 10-fold using Amicon Ultra 30K Centrifugal Filter Units (Merck Millipore Ltd).

### Enzyme linked immunosorbent assays (ELISA)

Nunc MaxiSorp flat-bottom 96-well plates were coated with 5 μg/mL of Galanthus nivalis Lectin (GNA-Lectin) (Sigma-Aldrich) and incubated overnight at 4°C. Plates were blocked with 4% skim milk in 1×PBS for one hour at RT or overnight at 4°C. Normalized amounts of E2 RBD glycoproteins were diluted in PBST (1×PBS with 0.05% TWEEN 20) (Sigma-Aldrich) and applied to the plates for three hours at RT or overnight at 4°C. After four washes of the plates with PBST, anti E2 or anti His-epitope tag antibodies were titrated across the plate and incubated for one hour at RT. Plates then washed 4 times with PBST. Bound antibodies to the E2 RBD glycoproteins were detected with rabbit anti-mouse or goat anti-rabbit antibodies conjugated with HRP for one hour at RT. ELISA plates were developed with TMB substrate (3,3’,5,5’-Tetramethylbenzidine dihydrochloride hydrate) (Sigma-Aldrich) dissolved in Phosphate-Citrate Buffer (Sigma-Aldrich) and stopped with 1M HCl. Optical density OD values at 450 nm subtracted from background OD level at 620 nm were obtained using a FLUOstar Optima microplate reader (BMG LabTechnologies, Germany). The ability of E2 RBD glycoproteins to bind a recombinant form of the large extracellular loop of CD81 (MBP-LEL^113–201^) was examined using ELISA as described previously [28].

### Western blotting

The expression of the E2 RBD glycoproteins was analysed by Western blotting (WB) or immunoprecipitation (IP) as previously described [6].

## Results

### Longitudinal sequence evolution in E2 RBD

**Fig 1:**
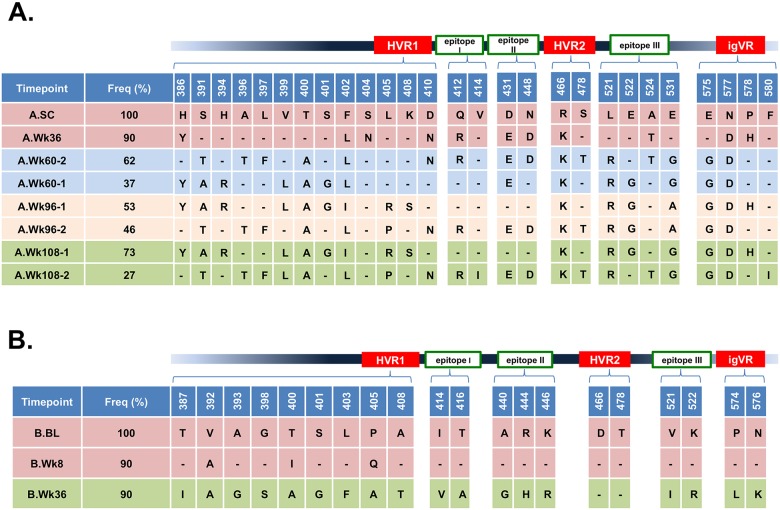
Amino acid changes within the E2 RBD from longitudinal samples of chronic and cleared patients. Samples of HCV infected patients were obtained from the Australian Trial in Acute Hepatitis C (ATAHC) prospective study. cDNA of the HCV E1E2 region was recovered from each timepoint of two patients (A) and (B). At least twenty clones were isolated from five and three timepoints of patients A and B, respectively. Sequences of each timepoint were aligned and amino acid differences within HVR1, HVR2, igVR, and epitopes I, II and III were marked with reference to the earliest timepoint available for each patient. Residue numbering is according to the H77c prototype sequence. SC = screening, BL = baseline.

Two individuals from the ATAHC cohort were selected for analysis in this study. One patient developed chronic HCV (patient A) while the other cleared his/her HCV infection after week 36 (patient B). Mutations in the E1E2 region amplified from longitudinal samples that resulted in amino acid changes deviating from the E1E2 sequence obtained at the earliest available time point are summarised in Fig 1 with full sequences of the E2 RBD region provided in S1 and S2 Figs. The overall dN/dS ratios in the E1E2 region for patient A and patient B were similar, 0.74 and 0.76, respectively. However, at individual sites within this region, stronger evidence of viral adaptation was apparent in patient A (S4 Table).

Within patient A, two distinct genotype 3a viral populations co-circulated from week 60 to week 108. Ninety percent of E1E2 clones obtained at 36 weeks after the initial screening timepoint (SC) comprised a single mutational signature indicating a dominant quasispecies (A.Wk36) (Fig 1A). At week 60, two distinct populations of viruses were observed with mutations present in 62% (A.Wk60-2) and 37% (A.Wk60-1) of the clones. By 96-weeks, the two viral populations were observed with similar frequencies of 53% (A.Wk96-1) and 46% (A.Wk96-2) of the clones, while mutations present in the latest time point examined, week 108, showed the two populations representing 73% (A.Wk108-1) and 27% (A.Wk108-2) of the clones (Fig 1A and S1 Fig). Within this patient, 2 amino acids in the E2 RBD region were under strong positive selection pressure as determined using two independent methods, L405 in HVR1 and L521 in epitope III (S4 Table).

In patient B, one dominant quasispecies had emerged by week 36 (Fig 1B and S2 Fig). In total, six mutations were found at week 8 (B.Wk8) and twenty one changes at week 36 (B.Wk36); three and 18 substitutions were located within the variable regions and/or CD81 binding sites of E2, respectively (Fig 1B and S2 Fig). HVR1 contained the highest number of mutations in both patients. In total, 13 sites of mutation evolved within HVR1 during the 108 weeks of HCV infection in patient A, with 17 mutations (15 unique) across 13 sites for A.Wk108-1 and 108–2 combined. In patient B, nine mutations were observed at 36 weeks. Comparison of the week 36 time point between patients reveals that the HVR1 region of the patient B had more mutations compared with patient A (Fig 1A and 1B) possibly suggesting that the HVR1 region was under stronger selection pressure in patient B. Indeed, in contrast to patient A, none of the E2 RBD residues in patient B had significant evidence of positive selection pressure by both of the codon-based methods applied here (S4 Table).

Neither HVR2 nor the igVR are known to be targets of the NAb response and the reason for sequence changes within these regions is unknown. While there were two amino acid changes within HVR2 of the patient A, no mutations were found within HVR2 of patient B (Fig 1A and 1B). Within patient A, the R466K mutation appeared at week 36 and was maintained until at least week 108. Interestingly, 4 mutations accumulated within the igVR of patient A, two of which, N577D and P578H, appeared at week 36. An additional two mutations appeared within the igVR; E575G was observed in both subpopulations from week 60, whereas F580I was only observed in one viral subpopulation at week 108. Of note, three mutations were present at all time points and in both viral populations in patient A; R466K within HVR2, and N577D within the igVR (Fig 1A) and at T495E located between HVR2 and igVR (S1 Fig). Within patient A, two igVR mutations occurred at week 36, P574L and N576K (Fig 1B and S2 Fig). The results indicate that the igVR is under significant selection pressure and contradicts earlier reports that variation only occurs between genotypes [6].

Evolutionary changes within the epitopes that overlap with the CD81 binding site were found in both patients. Changes adjacent to CD81 binding residues within the sequences amplified from patient A include mutations within epitope II at D431E and at the glycosylation site at N448 (Fig 1A and S1 Fig). Epitope III was found to be under considerable selection pressure in patient A with A524T present at week 36 between CD81 contact residues G523 and P525, and two additional mutations at E522G, adjacent to contact residue G523, and E531G/A, adjacent to G530 (Fig 1A and S1 Fig).

In patient B, 3 changes were observed within the CD81 binding region located in epitope II at A440G, R444H and K446R and a further 2 mutations within epitope III at V521I and K522R adjacent to CD81 contact residue G523 at week 36 (Fig 1B and S2 Fig).

### E2 RBD chimeras

**Fig 2:**
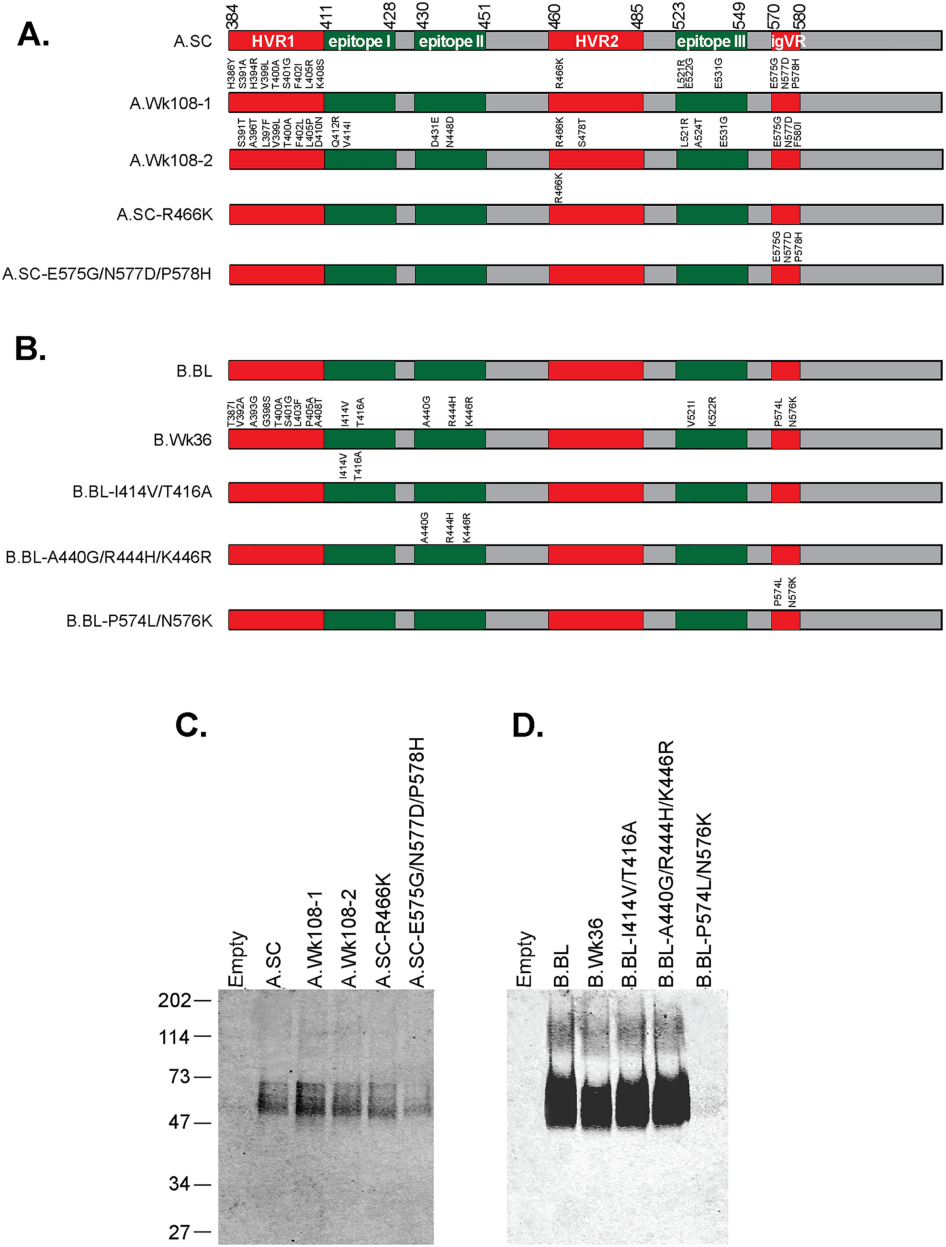
Expression of E2 RBD glycoproteins. (A) Schematic of E2 RBD constructs used in this study. The early timepoint A.SC and late timepoints A.Wk108-1 and A.Wk108-2 constructs were constructed from clones representing the dominant sequence of patient A at week 0 (SC) and 108 weeks. The A.SC-R466K chimera was constructed from the A.SC clone but contains a mutation within HVR2 at R466K. The A.SC-E575G/N577D/P578H chimera was constructed from the A.SC clone but contains mutations within the igVR at E575G, N577D, and P578H. Numbering is according to the prototype H77c sequence. (B) The B.BL and B.Wk36 constructs were prepared patient B consensus clones at BL and 36 weeks, respectively. The B.BL-I414V/T416A chimera was constructed from the B.BL clone but contains mutations I414V and T416A observed within epitope I. The B.BL-A440G/R444H/K446R chimera was constructed from the B.BL clone but contains mutations A440G, R444H, and K446R within the epitope II. The B.BL-P574L/N576K was constructed from the B.BL clone but contains mutations within the igVR region at P574L and N576K. The expression of E2 RBD glycoproteins of patient A (C) and patient B (D) derived from concentrated tissue culture fluid of transfected cells immunoprecipitated with anti E2 MAb24, separated using SDS-PAGE under reducing conditions and detected by Western blotting using anti His antibody. Empty = tissue culture fluid of mock transfected cells with pcDNA4c empty vector.

To further characterize how mutations within the E2 RBD affect E2 function, we examined their ability to bind the cell surface receptor CD81 and examined their antigenicity using E2 specific MAbs reactive to genotype 3a. Five E2 RBD clones were constructed from the longitudinal sequences of patient A (Fig 2A). A.SC is an RBD sequence amplified from a clone that represents the dominant viral population at the screening time point (S1 Fig). The first late time point chimera designated as A.Wk108-1 contained all mutations that appeared in the viral subpopulation that represented 73% of the sequences. The second late time point chimera referred to as A.Wk108-2 contained all mutations observed in the second viral subpopulation representing 27% of the sequences. Two additional E2 RBD clones were constructed to examine the effect of mutations found in HVR2 and the igVR. A single point mutation at R466K in HVR2 was observed in all clones of both viral subpopulations and was inserted into the E2 RBD A.SC sequence to give A.SC-R466K. Mutations observed within the igVR of the A.Wk108-1 subpopulation, E575G, N577D, and P578H were simultaneously inserted into the A.SC RBD sequence and named A.SC-E575G/N577D/P578H (Fig 2A).

To study the effect of sequence evolution in patient B, five E2 RBD clones were constructed (Fig 2B). B.BL RBD was derived from the dominant sequence observed at the baseline time point, whereas B.Wk36 RBD represents the dominant week 36 sequence (B.Wk36). To examine how mutations within the epitopes that overlap with CD81 binding regions affected glycoprotein function, two clones were constructed. The first clone included mutations in epitope I, I414V and T416A, and were inserted into the B.BL RBD clone (B.BL-I414V/T416V). The second clone contained epitope II mutations, A440G, R444H, and K446R, and were inserted into the B.BL RBD clone (B.BL-A440G/R444H/K446R). The mutations observed within the igVR, P574L and N576K, were inserted into the B.BL RBD clone (B.BL-P574L/N576K) (Fig 2B).

The E2 RBD glycoproteins were expressed and secreted from transfected HEK-293T cells at similar levels as detected by western blotting under reducing conditions (Fig 2C and 2D) except for B.BL-P574L/N576K E2 RBD, which was not detected with anti His antibody. As previously described, the E2 RBD glycoprotein appeared as diffuse ~50–60 kDa bands indicative of N-linked glycosylation [6].

The amounts of secreted E2 RBD glycoproteins present in transfection supernatants were normalized according to anti-His antibody reactivity in the western blot, in order that equivalent amounts of E2 RBD could be used in subsequent ELISA-based experiments, in which the RBDs are captured onto GNA-lectin coated or CD81 coated plates. The normalization method was validated when the majority of GNA-lectin captured E2 RBD proteins were detected at similar levels with anti His antibody and a MAb directed to the C-terminus of the E2 RBD (MAb26) (S3 Fig). Interestingly, the B.BL-P574L/N576K E2 RBD, which was not detected by anti-His antibody in western blot (Fig 2D), reacted with MAb26 in the ELISA, suggesting that the C-terminal His_6_ tag is occluded in the E2 RBD structure or that it is removed during expression. The data show that all E2 RBD glycoproteins were coated on plates at similar levels.

### Effect of E2 RBD sequence evolution on CD81 binding function

**Fig 3:**
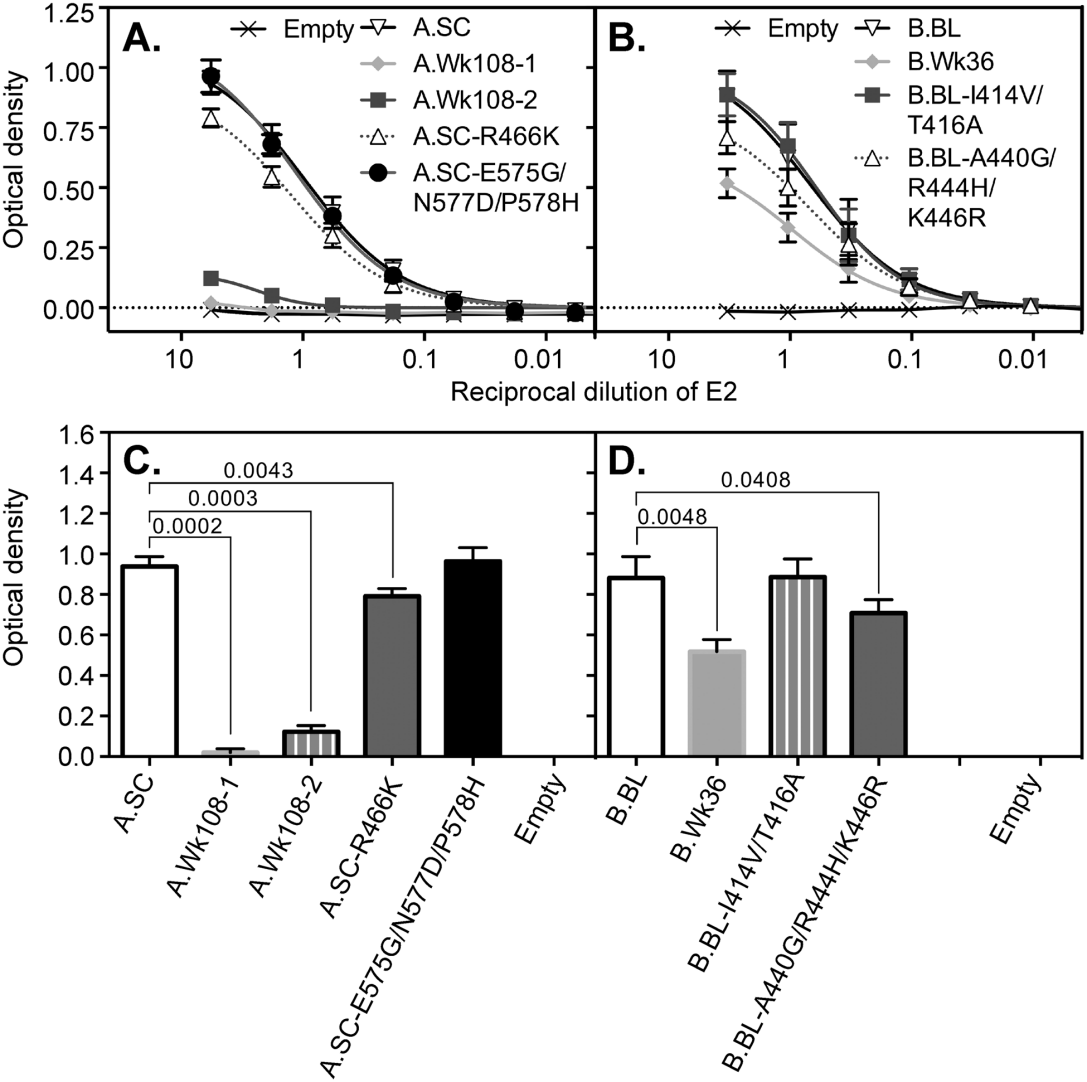
Ability of recombinant E2 RBD proteins to bind CD81. Normalized amounts of E2 RBD glycoproteins were applied to ELISA plates coated with the recombinant form of the CD81 large extracellular loop, MBP-LEL^113–201^. The E2 RBD glycoproteins from patient A (A) and patient B (B) were used in ELISA to detect binding to MBP-LEL^113–201^. Bound glycoproteins were detected with anti His antibody. Optical density was measured at 450 nm with background subtraction at 620 nm. The optical density at the second point of the curves is shown for patient A (C) and patient B (D) as means ± SEM from four independent experiments. *P* < 0.05 values were calculated by paired *t* test using GraphPad Prism 6 software.

To study the effect of E2 sequence evolution on CD81 binding ability, we employed a validated ELISA which measures the binding of solution-phase RBD to solid-phase recombinant dimeric CD81 large extracellular loop (MBP-LEL^113–201^) [9]. Normalized amounts of E2 RBD glycoproteins were applied to the ELISA plates coated with the CD81 recombinant protein MBP-LEL^113–201^ and RBD binding was detected with anti-His_6_ antibody. Results showed that the A.SC and the A.SC-E575G/N577D/P578H E2 RBD glycoproteins from patient A bound CD81 similarly (Fig 3A and 3C). However, the two E2 RBD glycoproteins constructed from the week 108 timepoints lacked CD81 binding ability (Fig 3A and 3C). This reduction in CD81 binding was partly attributable to the mutation R466K as this was sufficient to reduce the ability of the SC E2 RBD to bind CD81 (*P* = 0.0043) (Fig 3A and 3C). These results suggest that the CD81 binding site on the E2 RBD derived from patient A is inaccessible at week 108, but was not a result of mutations within the igVR and partly attributable to mutation within HVR2.

The CD81 binding ability of the RBD derived at the late point from patient B (B.Wk36) was reduced by ~ 50% relative to the baseline B.BL RBD (*P* = 0.0048), in concordance with the data obtained for patient A (Fig 3B and 3D). This reduction in binding could be largely attributed to 3 mutations in epitope II (A440G/R444H/K446R), which reduced CD81 binding by ~ 30% (*P* = 0.0408). By contrast, the epitope I I414V/T416A mutations (B.BL-I414V/T416A) had no effect. These results suggest that mutations present within the late time point sequences reduces the exposure of the CD81 binding site on E2 in patient B but is not a result of sequence changes in epitope-II alone.

#### Recognition of patient derived E2 RBD sequences by a broadly neutralizing antibody specific to epitope I

To examine whether recognition of the highly conserved neutralization epitope-I was affected by E2 sequence evolution during HCV infection, we employed a murine MAb specific to epitope-I and able to react with E2 RBDs from all 6 major genotypes and is broadly neutralizing (Alhammad *et al* submitted). The critical residues of MAb24 binding are L413, I414, N415, T416, G418, W420, and H421, with the latter two also being contact residues for CD81 binding [8, 10].

**Fig 4:**
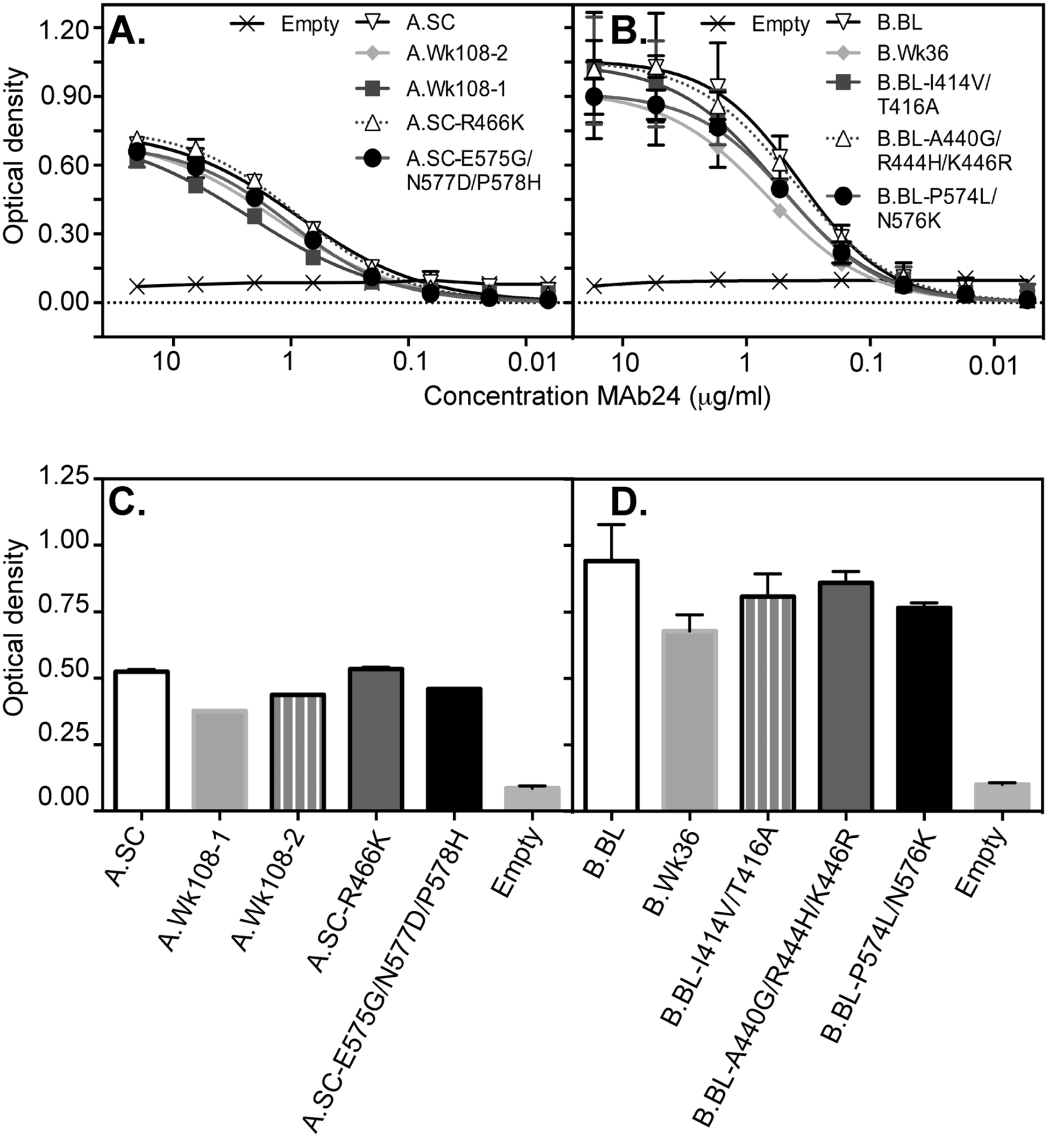
Binding of neutralizing MAb24 to E2 RBD glycoproteins. E2 RBD glycoproteins were normalized and applied to the ELISA plates coated with GNA-Lectin. Bound E2 RBD glycoproteins from (A) patient A and (B) patient B were detected with serially titrated neutralizing anti E2 MAb24 and secondary anti-mouse HRP antibodies. (C+D). The optical density at the third point of the curves is shown for patient A (C) and patient B in (D) as means ± SD from two independent experiments performed in duplicate.

The reactivity of early and late time point RBDs with MAb24 was compared in ELISA. MAb24 showed a small reduction in binding to the RBD representing the A.Wk108-1 viral population (Fig 4A and 4C) despite the absence of amino acid changes in epitope-I. (Fig 4A and 4C). The decreased MAb24 reactivity of A.Wk108-1 RBD was not attributable to R466K in HVR2 nor E575/N577D/P578H in the igVR suggesting a modest change in exposure of epitope I as a result of allosteric mutations elsewhere in the E2 RBD. By contrast, the MAb24 reactivity of A.Wk108-2 RBD was similar to that of A.SC even though two sequence changes (Q412R and I414V) occurred within epitope I distal to the CD81 contact residues, suggesting that this region is under selective pressure by epitope I specific antibodies.

In patient B, a slight reduction of MAb24 binding to B.Wk36 E2 RBD glycoprotein relative to binding to the B.BL E2 RBD was observed (Fig 4B and 4D). As in patient A, mutations within epitope I were present at week 36, I414V and T416A. As above, the I414V mutation reverts its epitope back to its preferred contact residue. However, T416A, in the context of a synthetic peptide, reduces MAb24 binding by approximately 50% (Alhammad *et al*, submitted). Indeed the binding of MAb24 to B.BL-I414V/T416A is lower than B.BL. Mutations within epitope II did not impact on MAb24 recognition, nor did changes in the igVR.

#### Longitudinal sequence evolution alters the exposure of non-neutralizing antibodies to epitope III

**Fig 5:**
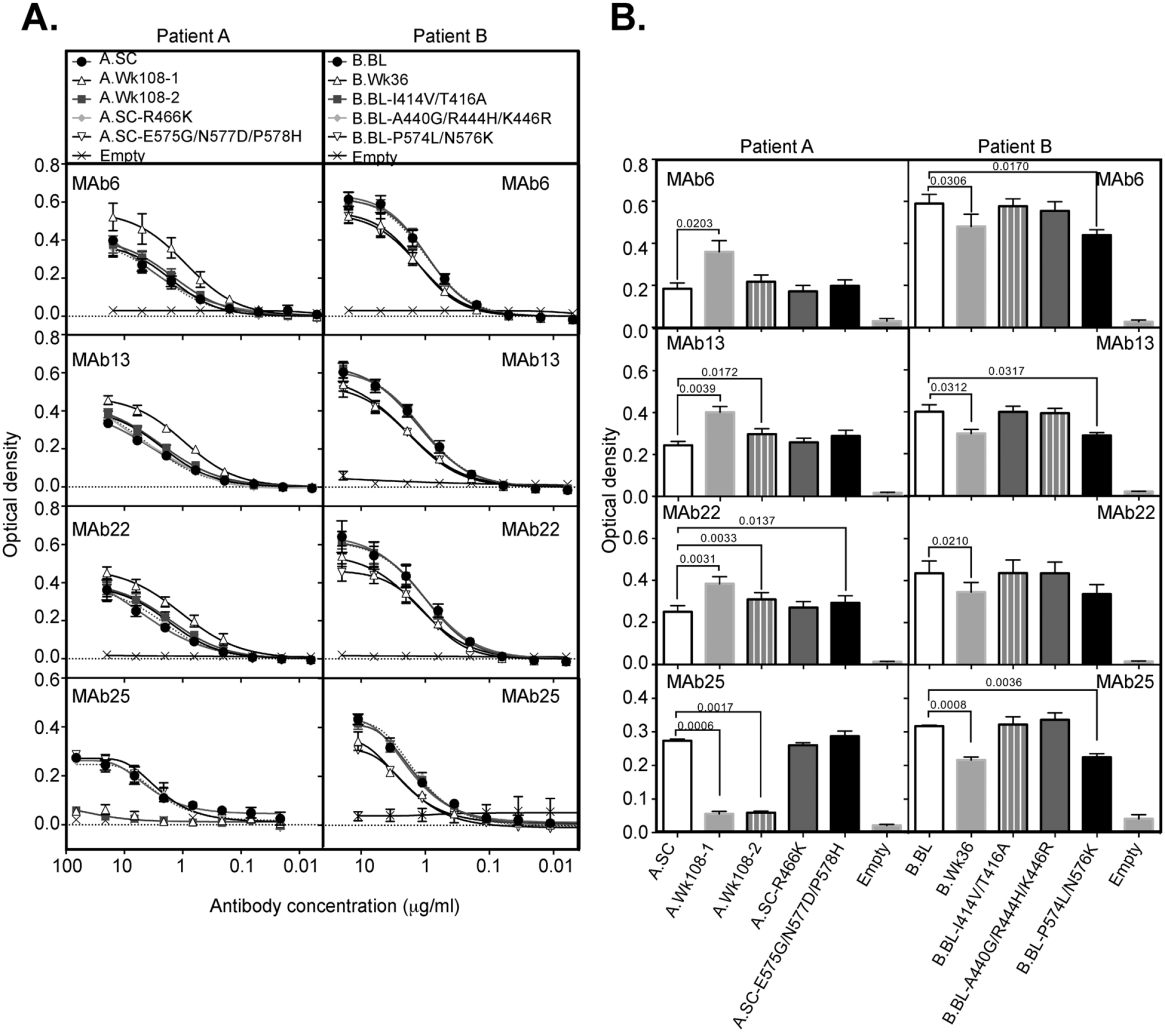
Binding of non-neutralizing MAbs to E2 RBD glycoproteins. E2 RBD glycoproteins were normalized and applied to the ELISA plates coated with GNA-Lectin. Bound E2 RBD glycoproteins were detected with anti E2 MAbs 6, 13, 22 and 25 and secondary anti-mouse HRP. The optical density at the second point of the curves is shown for the patient A and patient B as means ± SEM from three independent experiments. *P* < 0.05 values were calculated by paired *t* test using GraphPad Prism 6 software.

To further examine how sequence evolution impacts on E2 RBD structure and function, we used a panel of four genotype 3a reactive anti E2 MAbs; MAb6, MAb13, MAb22 and MAb25. All are specific for epitope III and all are non-neutralizing (Alhammad, *et al*. submitted) and do not inhibit E2-CD81 binding (Alhammad, *et al*. submitted). Examination of the viral sequences isolated from patients A and B revealed the known contact residues of these MAbs is completely conserved. MAbs 6, 13 and 22 bound more efficiently to the late A.Wk108-1 RBD suggesting greater exposure of their epitopes (Fig 5A and 5B) Amino acid changes within HVR2 or the igVR did not alter MAb 6 and 13 binding to epitope III (Fig 5A and 5B), whereas a small increase in MAb 22 binding was observed for the igVR combination mutant (Fig 5B; *P* = 0.0137). These results suggest that the accumulation of mutations in the E2 RBD of patient A leads to enhanced exposure of non-neutralizing epitopes within epitope III. By contrast, MAb25 failed to bind the E2 RBDs representing the two late time point subpopulations. In this case it is likely that either additional contact residues, not previously mapped, are contained within its epitope and are mutated in the late time point sequences, for example, at nearby positions 521 that is under strong selection pressure in patient A, 522 and 524 within its linear epitope, (Fig 1A). Alternatively, its epitope is completely occluded in the later time points as a result of mutations external to its epitope that alter presentation of the MAb25 epitope.

In patient B, MAbs 6, 13, 22 and 25 displayed significantly reduced binding to the late E2 RBD sequence, B.Wk36, but not to E2 RBDs containing mutations that accumulated within CD81 binding sites-I or II (B.BL-I414V/T416A or B.BL-A440G/R444H/K446R) (Fig 5B). However, mutations within the igVR when placed into the E2 RBD representing the earliest sequence (BL), B.BL-P574L/N576K, resulted in a significant reduction in the binding ability of MAbs 6, 13 and 25 (Fig 5B). These results indicate that in patient B, mutations within the igVR alter the exposure of epitope III, reducing accessibility by non-neutralizing antibodies.

## Discussion

In this study, longitudinal genotype 3a HCV viral sequence evolution from two patients infected with HCV was examined. Although genotype 3a represents approximately 30% of the HCV infections in the developed world, relatively little is known about genotype 3a E2 RBD structure or function. The E2 RBD region derived from the sequences within the two patients was used to examine how mutations within the E2 RBD affect exposure of the CD81 binding site and recognition of E2 RBD by neutralizing and non-neutralizing monoclonal antibodies.

Dominant sequences of five longitudinal samples from patient A and three samples from patient B were analysed (S1 and S2 Figs and Fig 1A and 1B). The largest number of amino acid changes occurred within the HVR1 of both patients with four and nine changes observed for patients A and B, respectively, within 36 weeks of infection indicating that sequence evolution was higher in the patient that cleared his/her infection than in the patient that developed chronic HCV in this time frame. Although our findings are limited to observations in two patients, they are in agreement with a previous study by Liu *et al* [18] that showed that the rate of HVR1 sequence evolution was higher in a patient that cleared HCV than in a patient that developed chronic infection. As HVR1 is a target of type-specific NAbs, it is likely that antibody driven selection pressure drives immune escape within HVR1 but appears to be more pronounced in patients that clear HCV. It is possible that an early type-specific NAb response to HVR1 is an important determinant of viral clearance possibly because these antibodies effectively control infection by preventing interaction of HCV through HVR1 with scavenger receptor class B type 1 [29–31].

The N448 residue is a conserved glycosylation site present in at least 97% of the prototypes sequences of the 7 HCV genotypes [21]. The removal of the N448 glycan from genotype 2a and 1a E2 resulted in enhanced binding to CD81, and enhanced neutralization by MAbs and HCV immune sera [20–22, 32]. These studies support the notion that glycosylation at N448 may shield the CD81 binding site from NAbs, contributing to immune evasion. In this study, the N448 glycan is ablated by a mutation in one of two subpopulations of HCV in patient A. However, both A.Wk108-1 (N448) and A.Wk108-2 (D448) showed markedly reduced binding to CD81 suggesting that the N448 glycan is perhaps not essential to CD81 binding for genotype 3a E2 RBDs. However, it is possible that other NAb epitopes may in fact be affected by the loss of glycan at N448. Additional studies with genotype 3a reactive antibodies are required to confirm this.

The reason why HVR2 and the igVR evolve during HCV infection is unclear. In this study, we show that the igVR is under considerable selection pressure as multiple mutations accumulated over time in both patients. Surprisingly, the igVR accumulated more sequence changes than the HVR2. Two and 4 mutations were observed within HVR2 and the igVR, respectively, in patient A. While no mutations occurred within HVR2 in patient B, two mutations occurred at week 36 within the igVR sequence (Fig 1A and 1B). The igVR was discovered by comparing cross-sectional sequences from the 6 available genotypes [6]. The finding here that the igVR mutates rapidly within infected patients suggests that it is in fact under considerable selection pressure, possibly more so than HVR2. Our analysis suggests that both may allosterically modulate CD81 binding and the exposure of antibody epitopes possibly providing an explanation for their evolution during infection.

It has been shown that HVR1 occludes the CD81 binding site in genotype 2a cell culture derived virions. Bankwitz et al proposed that the interaction of virions with host factors may then result in a conformational change that enhances the exposure of the CD81 binding site [7]. In this study, we found that the E2 RBD glycoproteins representing the week 108 timepoint of patient A (A.Wk108-1 and 2) lost the ability to bind to CD81, while the week 36 E2 RBD of patient B showed a 50% reduction in CD81 binding (Fig 3). A single mutation present within all sequences in HVR2 of patient A from week 36, R466K, slightly, but significantly reduced CD81 binding and contributed to the overall reduction in CD81 binding observed by E2 RBDs representing week 108 sequences (Fig 3A and 3C). In patient B, reduced binding of B.Wk36 E2 RBD to CD81 could be accounted by the A440G mutation, known to affect CD81 binding in genotype 1. The reduction in CD81 binding by these RBDs may be further attributed to occlusion of the CD81 binding site as a result of mutations external to those directly involved in CD81 binding. In both patients, mutations accumulated within three epitopes that overlap with the three major discontinuous CD81 binding regions (Fig 1). Alternatively, mutations within HVR1 alone or in combination with other mutations, may alter the conformation of the CD81 binding site such that it is occluded in the later time points. The progressive occlusion of the CD81 binding site during HCV infection would provide HCV with the ability to shield itself from the adaptive immune response to lessen the host’s ability to elicit NAbs directed to the CD81 binding site. In addition, early in infection, there may be a greater dependence on E2-CD81 interactions such that only viruses with high-affinity and multivalent interactions are selected [33]. As infection progresses, the dependence on CD81 is reduced and viral subpopulations with low affinity binding are selected because they allow entry but would simultaneously make this region a subdominant neutralization epitope.

The antigenicity of the E2 RBDs towards epitopes I, II and III was examined for both patients. The binding of cross-reactive non-neutralizing antibodies directed towards epitope III, MAbs 6, 13, and 22, was increased in the A.Wk108-1 E2 RBD glycoprotein (Fig 5). The known contact residues recognised by these antibodies is completely conserved in these E2 RBDs and suggests that mutations in E2 alter the conformation of epitope III and increases exposure of non-neutralizing epitopes. Changes within the HVR1 or elsewhere in E2, but not HVR2 or the igVR, could contribute to the enhancement of binding by these MAbs in patient A. In contrast, the binding of non-neutralizing antibodies to epitope III was reduced in the patient B and could be directly ascribed to mutations in the igVR. In this case, the evolution of the igVR may have occluded non-neutralizing antibody epitopes thereby favouring the elicitation of NAbs. Further studies examining how the igVR regulates exposure of NAbs in patients with different clinical outcomes is required to confirm this observation.

The binding of a neutralizing antibody specific to epitope I (MAb24) was slightly reduced in late time point sequences of both patients. Epitope I is recognized by broadly neutralizing human MAbs, AR1A, HC33, 95–2 and HCV-1 [34–36]. Within the epitope I sequence of both patients studied here, mutations occurred at Q412R, V414I, I414V and T416A. The presence of mutations at I414, and T416 in both patients suggests that this region is under direct selection pressure during infection most likely as a result of the NAb response. Recent crystallographic studies of neutralizing monoclonal antibodies with peptides representing epitope I reveal that this region is highly flexible and can adopt alternate conformations induced by the antibody [12–14]. This structural flexibility may also favour immune evasion as the region does not appear to be constrained to adopt a fixed conformation to maintain E2 structure or function.

**Fig 6:**
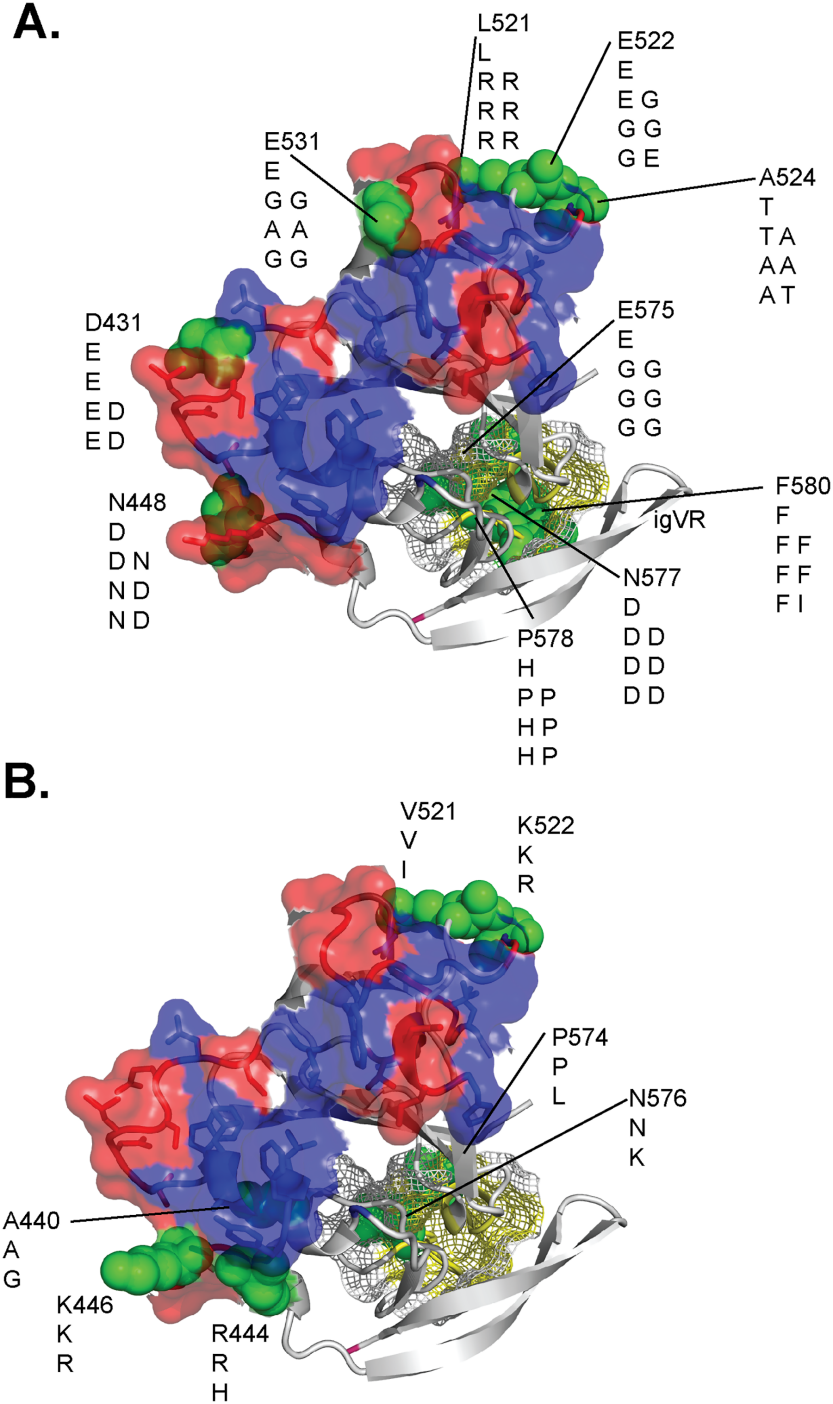
Location of mutations on the E2 RBD structure. The structure of the E2 core domain was obtained from [5]. The neutralizing face of E2 is shown in red and the CD81 contact residues as described by Kong et al [5] and Drummer et al [9] is shown in blue. The non-neutralizing face is shown in gray and the igVR is shown in yellow. Longitudinal sequence changes observed in the patient A (A) and patient B (B) were mapped on the E2 structure and indicated as green spheres. The amino acid mutation and position are labelled and the longitudinal mutations indicated. For patient A where two distinct viral sequences were observed at weeks 60, 96 and 108, the amino acid changes observed in the major and minor sub-populations are shown side-by-side, respectively.

Recently the crystal structure of the E2 RBD was solved in association with MAbs specific to epitope III [4, 5]. The genotype 1a HCV E2 structure of Kong *et al* (2013) has an immunoglobulin-like fold but lacks information for HVR1, the neutralization epitope-I, 411–421, and HVR2. In addition, two glycosylation sites were mutated at N448 and N576 [5]. The early time-point sequences of both patients differ at 47 positions of the 212 in the E2 core domain structure (78% identity). However, it is likely that the overall fold of the E2 region is highly conserved and allows us to model the likely positions of the amino acid changes that occur in each of the patients in relation to know CD81 contact residues. In Fig 6 we have modelled the location of the dominant mutations present at late time points on the genotype 1a E2 structure [5]. A distinct pattern of mutational evolution can be observed in the two patients, with a higher frequency of mutations clustering around the CD81 binding site observed for patient A than in patient B (Fig 6A and 6B). The majority of the mutations in both patients are located on the neutralizing face of E2 but not at residues known to be directly involved in CD81 binding (Fig 6A and 6B). These data suggest that acquisition of NAbs towards the conserved core domain of E2 drives immune escape but preserves the CD81 contact residues. Our data suggest that allosteric changes to the E2 RBD may occur to occlude the CD81 binding site or reduce its affinity of binding such that we can no longer detect interactions in solid phase binding experiments. Such a mechanism would likely preclude development of potent NAbs whose mechanism is to directly prevent contact with CD81. However, it is likely that CD81 binding will still occur in the virions but may require conformational changes to reveal the CD81 binding site or low affinity interactions suffice to initiate entry.

### Limitations

There are several limitations to this study. Firstly, it was not possible to obtain infectious pseudotyped retroviral particles from the E1E2 envelope proteins obtained from either patient at any time point precluding an analysis of how evolution relates to infectivity. As such we were restricted to an analysis of the E2 RBD regions. Second, limited numbers of antibodies recognise genotype 3a E2 and restricted the analysis to those used in this study. Third, solid-phase binding assays were employed to study interactions between E2 and CD81. Whilst we have extensively used and characterized this method, it is accepted that the affinity of virion incorporated E2 to the intact CD81 tetraspanin is likely to be much higher than that of recombinant E2 for recombinant CD81 LEL whose affinity is in the low nM range. Therefore, we would expect our solid phase assay to be a more sensitive measure of changes in E2-CD81 and that some mutations may not dramatically affect virion-CD81 interactions. Finally, our analysis was restricted to two patients with different clinical outcomes. Nevertheless this is the first to study of sequence evolution within the E2 region and the consequences on CD81 binding and antigenicity in genotype 3a infection. Future studies on larger numbers of patients may be able to attribute whether the rate of evolution and exposure/occlusion of antibody epitopes and the CD81 binding site correlate with clinical outcomes.

The overall findings of this study suggest that the E2 RBD evolves considerably during infection to modulate the exposure of the CD81 binding sites and both neutralizing- and non-neutralizing antibody epitopes. This was partly attributable to mutations within the HVR2 and the igVR and suggested that these regions allosterically modulate exposure of E2 epitopes.

## Supporting Information

S1 Fig Alignment of amino acid sequences within the E2 RBD region from longitudinal samples collected from patient A. The cDNA of the viral E1E2 region was recovered from each timepoint. The consensus sequences of twenty clones were isolated and aligned from five timepoints. Amino acid substitutions from the earliest timepoint (A.SC) were compared to later timepoints isolated at week 36 (A.36Wk-1 and 2), week 60 (A.60Wk-1 and 2), week 96 (A.96Wk-1 and 2), and week 108 (A.Wk108-1 and 2). Bold residues in the SC timepoint are residues where mutations occurred in later timepoints, highlighted in red. Numbering is according to the prototype H77c sequence. SC = screening. The location of HVR1, HVR2 and the igVR are indicated as are residues involved in CD81 binding corresponding to those highlighted in Fig 6 (gray), and epitopes I, II and III (underlined) on the H77c sequence.
(PDF)

S2 Fig Alignment of longitudinal amino acid sequences within the E2 RBD region of patient B. The cDNA of the viral E1E2 region was recovered from each timepoint. The consensus sequences of twenty clones were isolated and aligned from three timepoints. Amino acid substitutions from the earliest timepoint were (B.BL) compared to later timepoints at week 8 (B.Wk8) and week 36 (B.Wk36). Bold residues in the BL timepoint are residues where mutations occurred in later timepoints, highlighted in red Numbering is according to the prototype H77c sequence. SC = screening. The location of HVR1, HVR2 and the igVR are indicated as are residues involved in CD81 binding corresponding to those highlighted in Fig 6 (gray), and epitopes I, II and III (underlined) on the H77c sequence. BL = baseline.
(PDF)

S3 Fig Chimeric E2 RBD glycoproteins captured with GNA-Lectin. Normalized amounts of E2 RBD glycoproteins were applied to ELISA plates coated with GNA-Lectin. Bound E2 RBD glycoproteins from patient A (A and C) and patient B (B and D) to GNA-Lectin were detected with serially titrated anti His antibody (A and B) or anti E2 MAb26 (C and D). Optical density (OD) was measured at 450 nm with background subtraction at 620 nm. The results show that equivalent amounts of E2 protein were applied to the ELISA plates.
(PDF)

S1 Table Sample information of HCV infected patients. (PDF)

S2 Table Primers used to amplify the region encoding E1E2. (PDF)

S3 Table Primers used to amplify E2 RBD constructs. (PDF)

S4 Table dN/dS analysis of sequences derived form this study. (PDF)
