# Supplementary material for: Longitudinal Sequence and Functional Evolution within Glycoprotein E2 in Hepatitis C Virus Genotype 3a Infection
Source: PLoS One. 2015 May 13;10(5):e0126397. doi: 10.1371/journal.pone.0126397 (PMC4430534; doi:10.1371/journal.pone.0126397)
Supplement: S1 Fig — The cDNA of the viral E1E2 region was recovered from each timepoint. The consensus sequences of twenty clones were isolated and aligned from five timepoints. Amino acid substitutions from the earliest timepoint (A.SC) were compared to later timepoints isolated at week 36 (A.36Wk-1 and 2), week 60 (A.60Wk-1 and 2), week 96 (A.96Wk-1 and 2), and week 108 (A.Wk108-1 and 2). Bold residues in the SC timepoint are residues where mutations occurred in later timepoints, highlighted in red. Numbering is according to the prototype H77c sequence. SC = screening. The location of HVR1, HVR2 and the igVR are indicated as are residues involved in CD81 binding corresponding to those highlighted in Fig 6 (gray), and epitopes I, II and III (underlined) on the H77c sequence. (PDF) [file pone.0126397.s001.pdf]

```
              ▶384     HVR1                       Epitope I         Epitope II
H77c          ETHVTGGNAGRTTAGLVGLLTPGAKQNIQLINTNGSWHINSTALNCNESLNTGWLAGLFY 443
A.SC  (100%)  STHVTGGSAAHGALGVTSFFSLGPKQDLQLVNTNGSWHINRTALNCNDSINTGFIAGLFY 443
A.Wk36 (90%)  STYVTGGSAAHGTLGVTSLFNLGPKQNLRLVNTNGSWHINRTALNCNESINTGFIAGLFY 443
A.Wk60-1 (62%) STHVTGGTAAHGTFGVASLFSLGPKQNLRLVNTNGSWHINRTALNCNESINTGFIAGLFY 443
A.Wk60-2 (37%) STYVTGGAAARGALGLAGLFSLGPKQDLQLVNTNGSWHINRTALNCNESINTGFIAGLFY 443
A.Wk96-1 (53%) STYVTGGAAARGALGLAGIFSRGPSQDLQLVNTNGSWHINRTALNCNDSINTGFIAGLFY 443
A.Wk96-2 (46%) STHVTGGTAAHGTFGVASLFSPGPKQNLRLVNTNGSWHINRTALNCNESINTGFIAGLFY 443
A.Wk108-1(73%) STYVTGGAAARGALGLAGIFSRGPSQDLQLVNTNGSWHINRTALNCNDSINTGFIAGLFY 443
A.Wk108-2(27%) STHVTGGTAAHGTFGVASLFSPGPKQNLRLINTNGSWHINRTALNCNESINTGFIAGLFY 443

                                     HVR2
H77c          QHKFNSSGCPERLASCRRLTDFAQGWGPISYANGSGLDE-RPYCWHYPPRPCGIVPAKSV 502
A.SC  (100%)  YNKFNSTGCPQRLSSCKPITSFRQGWGSLTDANISGPSEDKPYCWHYAPRPCTIVPASSV 503
A.Wk36 (90%)  YNKFDSTGCPQRLSSCKPITSFKQGWGSLTDANISGPSEDKPYCWHYAPRPCEIVPASSV 503
A.Wk60-1 (62%) YNKFDSTGCPQRLSSCKPITSFKQGWGSLTDANITGPSEDKPYCWHYAPRPCEIVPASSV 503
A.Wk60-2 (37%) YNKFNSTGCPQRLSSCKPITSFKQGWGSLTDANISGPSEDKPYCWHYAPRPCEIVLASSV 503
A.Wk96-1 (53%) YNKFNSTGCPQRLSSCKPITSFKQGWGSLTDANISGPSEDKPYCWHYAPRPCEIIPASSV 503
A.Wk96-2 (46%) YNKFDSTGCPQRLSSCKPITSFKQGWGSLTDANITGPSEDKPYCWHYAPRPCEIVPASSV 503
A.Wk108-1(73%) YNKFNSTGCPQRLSSCKPITSFKQGWGSLTDANISGPSEDKPYCWHYAPRPCEIIPASSV 503
A.Wk108-2(27%) YNKFDSTGCPQRLSSCKPITSFKQGWGSLTDANITGPSEDKPYCWHYAPKPCEIVPASSV 503

                                        Epitope III
H77c          CGPVYCFTPSPVVVGTTDRSGAPTYSWGANDTDVFVLNNTRPPLGNWFGCTWMNSTGFTK 562
A.SC  (100%)  CGPVYCFTPSPVVVGTTDLEGAPTYTWGENETDVFLLESLRPPSGRWFGCTWMNSTGFTK 563
A.Wk36 (90%)  CGPVYCFTPSPVVVGTTDLEGTPTYTWGENETDVFLLESLRPPSGRWFGCTWMNSTGFTK 563
A.Wk60-1 (62%) CGPVYCFTPSPVVVGTTDREGTPTYTWGGNETDVFLLESLRPPSGRWFGCTWMNSTGFTK 563
A.Wk60-2 (37%) CGPVYCFTPSPVVVGTTDRGGAPTYTWGGNETDVFLLESLRPPSGRWFGCTWMNSTGFTK 563
A.Wk96-1 (53%) CGPVYCFTPSPVVVGTTDRGGAPTYTWGANETDVFLLESLRPPSGRWFGCTWMNSTGFTK 563
A.Wk96-2 (46%) CGPVYCFTPSPVVVGTTDRGGAPTYTWGANETDVFLLESLRPPSGRWFGCTWMNSTGFTK 563
A.Wk108-1(73%) CGPVYCFTPSPVVVGTTDRGGAPTYTWGGNETDVFLLESLRPPSGRWFGCTWMNSTGFTK 563
A.Wk108-2(27%) CGPVYCFTPSPVVVGTTDREGTPTYTWGGNETDVFLLESLRPPSGRWFGCTWMNSTGFTK 563

                    IgVR
H77c          VCGAPPCVI-GGVG---N-NTLLCPTDCFRKHPEATYSRCGSGPWITPRCMVDYPYRLWH 617
A.SC  (100%)  TCGAPPCNIYGGEGNPANDSDLFCPTDCFRKHPEATYSRCGAGPWLTPRCLVDYPYRLWH 623
A.Wk36 (90%)  TCGAPPCNIYGGEGDHANDSDLFCPTDCFRKHPEATYSRCGAGPWLTPRCLVDYPYRLWH 623
A.Wk60-1 (62%) TCGAPPCNIYGGGGDPANDSDLFCPTDCFRKHPEATYSRCGAGPWLTPRCLVDYPYRLWH 623
A.Wk60-2 (37%) TCGAPPCNIYGGGGDPANDSDLFCPTDCFRKHPEATYSRCGAGPWLTPRCLVDYPYRLWH 623
A.Wk96-1 (53%) TCGAPPCNIYGGGGDHANDSDLFCPTDCFRKHPEATYSRCGAGPWLTPRCLVDYPYRLWH 623
A.Wk96-2 (46%) TCGAPPCNIYGGGGDPANDSDLFCPTDCFRKHPEATYSRCGAGPWLTPRCLVDYPYRLWH 623
A.Wk108-1(73%) TCGAPPCNIYGGGGDHANDSDLFCPTDCFRKHPEATYSRCGAGPWLTPRCLVDYPYRLWH 623
A.Wk108-2(27%) TCGAPPCNIYGGGGDPANDSDLICPTDCFRKHPEATYSRCGAGPWLTPRCLVDYPYRLWH 623

H77c          YPCTINYTIFKVRMYVGGVEHRLEAACNWTRGERCDLEDRDRSE 661
A.SC  (100%)  YPCTINFTIFKVRTFVGGFEHRFDAACNWTRGERCNIEDRDRSE 668
A.Wk36 (90%)  YPCTVNFTIFKVRTFVGGFEHRFDAACNWTRGERCNIEDRDRSE 668
A.Wk60-1 (62%) YPCTINFTIFKVRTFVGGFEHRFDAACNWTRGERCNIEDRDRSE 668
A.Wk60-2 (37%) YPCTINFTIFKVRTFVGGFEHRFDAACNWTRGERCNIEDRDRSE 668
A.Wk96-1 (53%) YPCTINFTIFKVRTFVGGFEHRFDAACNWTRGERCNIEDRDRSE 668
A.Wk96-2 (46%) YPCTINFTIFKVRTFVGGFEHRFDAACNWTRGERCNIEDRDRSE 668
A.Wk108-1(73%) YPCTVNFTIFKVRTFVGGFEHRFDAACNWTRGERCNIEDRDRSE 668
A.Wk108-2(27%) YPCTINFTIFKVRTFVGGFEHRFDAACNWTRGERCNIEDRDRSE 668
```

S1 Fig. Alignment of amino acid sequences within the E2 RBD region from longitudinal samples collected from patient A. The cDNA of the viral E1E2 region was recovered from each timepoint. The consensus sequences of twenty clones were isolated and aligned from five timepoints. Amino acid substitutions from the earliest timepoint (A.SC) were compared to later timepoints isolated at week 36 (A.36Wk-1 and 2), week 60 (A.60Wk-1 and 2), week 96 (A.96Wk-1 and 2), and week 108 (A.Wk108-1 and 2). Bold residues in the SC timepoint are residues where mutations occurred in later timepoints, highlighted in red. Numbering is according to the prototype H77c sequence. SC = screening. The location of HVR1, HVR2 and the igVR are indicated as are residues involved in CD81 binding corresponding to those highlighted in Figure 6 (gray), and epitopes I, II and III (underlined) on the H77c sequence.
