# Supplementary material for: Longitudinal Sequence and Functional Evolution within Glycoprotein E2 in Hepatitis C Virus Genotype 3a Infection
Source: PLoS One. 2015 May 13;10(5):e0126397. doi: 10.1371/journal.pone.0126397 (PMC4430534; doi:10.1371/journal.pone.0126397)
Supplement: S2 Fig — The cDNA of the viral E1E2 region was recovered from each timepoint. The consensus sequences of twenty clones were isolated and aligned from three timepoints. Amino acid substitutions from the earliest timepoint were (B.BL) compared to later timepoints at week 8 (B.Wk8) and week 36 (B.Wk36). Bold residues in the BL timepoint are residues where mutations occurred in later timepoints, highlighted in red Numbering is according to the prototype H77c sequence. SC = screening. The location of HVR1, HVR2 and the igVR are indicated as are residues involved in CD81 binding corresponding to those highlighted in Fig 6 (gray), and epitopes I, II and III (underlined) on the H77c sequence. BL = baseline. (PDF) [file pone.0126397.s002.pdf]

```
                          ▶384       HVR1                      Epitope I        Epitope II
H77c                      ETHVTGGNAGRTTAGLVGLLTPGAKQNIQLINTNGSWHINSTALNCNESLNTGWLAGLFY 443

B.BL    (100%)            TTYTSGGTVAYNTRGLTSLLSPGPAQNLQLINTNGSWHINRTALNCNDSLNTGFIAALFY 443
B.Wk8    (90%)            TTYTSGGTAAYNTRGLISLLSQGPAQNLQLINTNGSWHINRTALNCNDSLNTGFIAALFY 443
B.Wk36   (90%)            TTYISGGTAGYNTRSLAGLFSAGPTQNLQLVNANGSWHINRTALNCNDSLNTGFIAGLFY 443

                                                     HVR2
H77c                      QHKFNSSGCPERLASCRRLTDFAQGWGPISYANGSG-LDERPYCWHYPPRPCGIVPAKSV 502

B.BL    (100%)            RYKFNSTGCPERLSSCKPITFFDQGWGPLTDTNITGSSDDKPYCWHYAPRLCNIVPAANV 503
B.Wk8    (90%)            RYKFNSTGCPERLSSCKPITFFDQGWGPLTDTNITGSSDDKPYCWHYAPRPCNIVPAANV 503
B.Wk36   (90%)            HYRFNSTGCPERLSSCKPITFFDQGWGPLTDTNITGSSDDKPYCWHYAPRLCKIVPAANV 503

                                            Epitope III
H77c                      CGPVYCFTPSPVVVGTTDRSGAPTYSWGANDTDVFVLNNTRPPLGNWFGCTWMNSTGFTK 562

B.BL    (100%)            CGPVYCFTPSPVVVGTTDVKGAPTYTWGENETDVFLLESLRPPSGRWFGCVWMNSTGFVK 563
B.Wk8    (90%)            CGPVYCFTPSPVVVGTTDVKGAPTYTWGENETDVFLLESLRPPSGRWFGCVWMNSTGFVK 563
B.Wk36   (90%)            CGPVYCFTPSPVVVGTTDIRGAPTYTWGENETDVFLLESLRPPSGRWFGCVWMNSTGFVK 563

                                    igVR
H77c                      VCGAPPCVIGG----VGNN--TLLCPTDCFRKHPEATYSRCGSGPWITPRCMVDYPYRLW 616

B.BL    (100%)            TCGAPPCNIYGDRRGPSNNGTTLFCPTDCFRKHPDATYSRCGAGPWLTPRCLVDYPYRLW 623
B.Wk8    (90%)            TCGAPPCNIYGDRRGPSNNGTTLFCPTDCFRKHPDATYSRCGSGPWLTPRCLVDYPYRLW 623
B.Wk36   (90%)            TCGAPPCNIYGDRRGLSKNGTTLFCPTDCFRKHPDATYSRCGAGPWLTPRCLVDYPYRLW 623


H77c                      HYPCTINYTIFKVRMYVGGVEHRLEAACNWTRGERCDLEDRDRSE 661

B.BL    (100%)            HYPCTVNFTLFKVRMFVGGFEHRFTAACNWTRGERCDIEDRDRSE 668
B.Wk8    (90%)            HYPCTVNFTLFKVRMFVGGFEHRFTAACKWTRGERCDIEDRDRSE 668
B.Wk36   (90%)            HYPCTVNFTLFKVRMFVGGFEHRFTAACNWTRGDPCDIEDRDRSE 668
```

S2 Fig. Alignment of longitudinal amino acid sequences within the E2 RBD region of patient B. The cDNA of the viral E1E2 region was recovered from each timepoint. The consensus sequences of twenty clones were isolated and aligned from three timepoints. Amino acid substitutions from the earliest timepoint were (B.BL) compared to later timepoints at week 8 (B.Wk8) and week 36 (B.Wk36). Bold residues in the BL timepoint are residues where mutations occurred in later timepoints, highlighted in red Numbering is according to the prototype H77c sequence. SC = screening. The location of HVR1, HVR2 and the igVR are indicated as are residues involved in CD81 binding corresponding to those highlighted in Figure 6 (gray), and epitopes I, II and III (underlined) on the H77c sequence. BL = baseline.
