# Supplementary material for: Longitudinal Sequence and Functional Evolution within Glycoprotein E2 in Hepatitis C Virus Genotype 3a Infection
Source: PLoS One. 2015 May 13;10(5):e0126397. doi: 10.1371/journal.pone.0126397 (PMC4430534; doi:10.1371/journal.pone.0126397)
Supplement: S3 Fig — Normalized amounts of E2 RBD glycoproteins were applied to ELISA plates coated with GNA-Lectin. Bound E2 RBD glycoproteins from patient A (A and C) and patient B (B and D) to GNA-Lectin were detected with serially titrated anti His antibody (A and B) or anti E2 MAb26 (C and D). Optical density (OD) was measured at 450 nm with background subtraction at 620 nm. The results show that equivalent amounts of E2 protein were applied to the ELISA plates. (PDF) [file pone.0126397.s003.pdf]

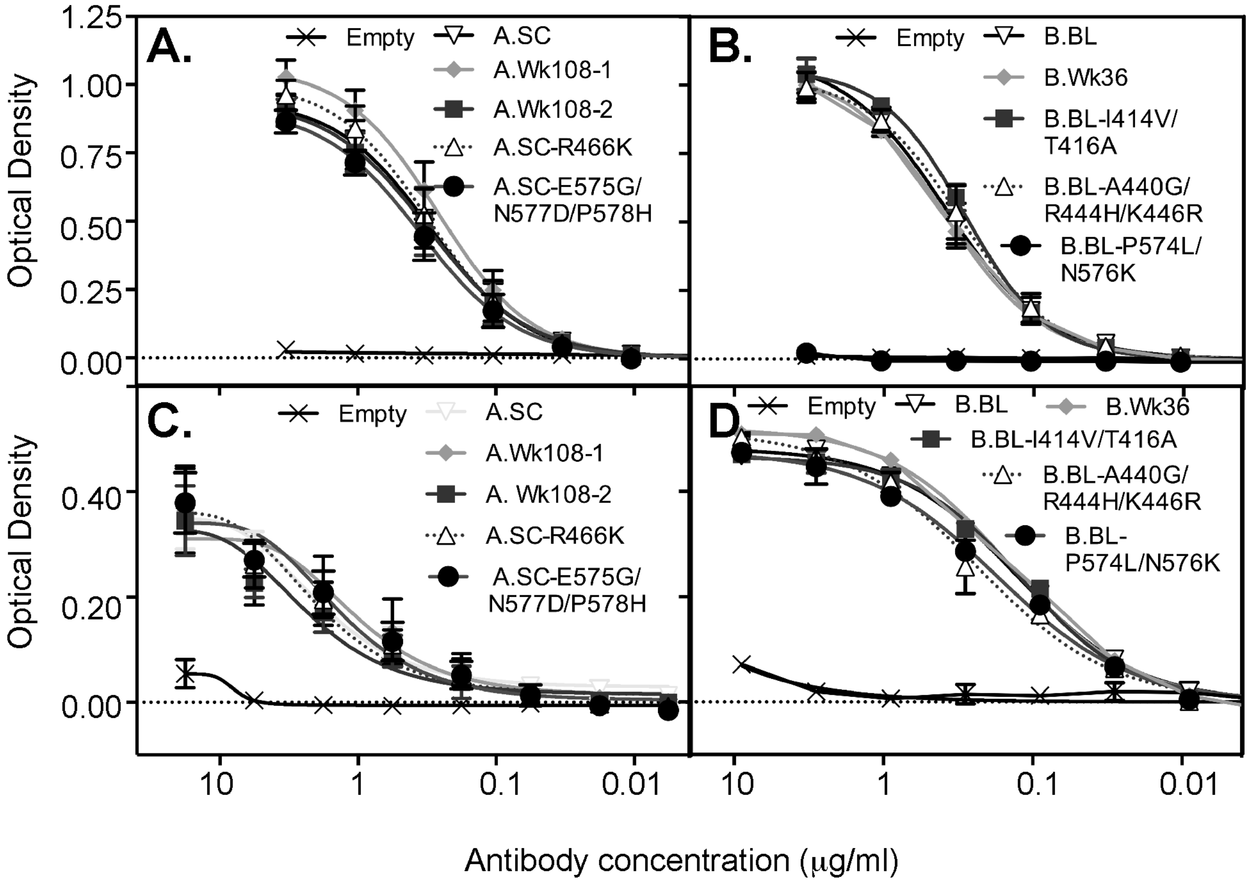

S3 Fig. Chimeric E2 RBD glycoproteins captured with GNA-Lectin. Normalized amounts of E2 RBD glycoproteins were applied to ELISA plates coated with GNA-Lectin. Bound E2 RBD glycoproteins from patient A (A and C) and patient B (B and D) to GNA-Lectin were detected with serially titrated anti His antibody (A and B) or anti E2 MAb26 (C and D). Optical density (OD) was measured at 450 nm with background subtraction at 620 nm. The results show that equivalent amounts of E2 protein were applied to the ELISA plates.
