# Supplementary material for: Longitudinal Sequence and Functional Evolution within Glycoprotein E2 in Hepatitis C Virus Genotype 3a Infection
Source: PLoS One. 2015 May 13;10(5):e0126397. doi: 10.1371/journal.pone.0126397 (PMC4430534; doi:10.1371/journal.pone.0126397)
Supplement: S1 Table — (PDF) [file pone.0126397.s004.pdf]

S1 Table. Sample information of HCV infected patients.

Patient	Week	Collection date	CV quantitative[1]	Genotype[1]	E1/E2 [2]
**Patient A**	SC	26/10/2004	13 000 000	3a	1
	36	21/09/2005	2 400 000		1
	60	4/04/2006	3 246 618		1
	96	22/11/2006	6 750 889		1
	108	26/02/2007	7 388 034		1
**Patient B**	SC	18/01/2006	11 000	3a	0
	BL	16/02/2006	89 230		1
	8	10/05/2006	206 268		1
	24	2/08/2006	503 200		0
	36	25/11/2006	236 486		1

SC screening. BL Baseline

1. Viral load IU/ml. A quantitative assay was performed using the Versant HCV RNA 3.0 Bayer with a lower limit of detection 615 IU/mL and HCV genotyping was performed using a commercial assay (Versant LiPa2, Bayer).
2. RT-PCR result. 1= product amplified. 0=no product amplified.
