# Supplementary material for: Longitudinal Sequence and Functional Evolution within Glycoprotein E2 in Hepatitis C Virus Genotype 3a Infection
Source: PLoS One. 2015 May 13;10(5):e0126397. doi: 10.1371/journal.pone.0126397 (PMC4430534; doi:10.1371/journal.pone.0126397)
Supplement: S2 Table — (PDF) [file pone.0126397.s005.pdf]

S2 Table. Primers used to amplify the region encoding E1E2.

Primers	Sense – purpose	Sequence
GV32	1st strand – Outer forward primer binds to C-terminus of Core	5'- TGG GTA ARG TCA TCG ATA CCC T-3'
GV34	2nd strand – Inner forward primer binds to C-terminus of Core	5'- TGC GGM TTC GCC GAC CTC ATG G-3'
GV150	1st strand – Outer reverse primer binds NS2	5'- CAT ATR AGG CGG CCG ATC-3'
GV151	2nd strand – Inner reverse primer binds NS2	5'- CCG AGA GTR GCG CTG TCT T-3'
