# Supplementary material for: Longitudinal Sequence and Functional Evolution within Glycoprotein E2 in Hepatitis C Virus Genotype 3a Infection
Source: PLoS One. 2015 May 13;10(5):e0126397. doi: 10.1371/journal.pone.0126397 (PMC4430534; doi:10.1371/journal.pone.0126397)
Supplement: S3 Table — (PDF) [file pone.0126397.s006.pdf]

S3 Table. Primers used to amplify E2 RBD constructs.

Primers	Sense – purpose	Sequence
Nhe1.E2.FWD	Forward primer to amplify early and late time points in both patients	5'- CGC GCTAGC AGT ACA CAT GTC ACC GGT GG-3'
XbaI.E2R.His	Reverse primer to amplify early and late time points with a His-epitope tag in both patients	5'- CCG TCT AGA TTA GTG GTG GTG GTG GTG GTG CTC GCT GCG GTC GCG GTC CTC GAT-3'
NheI.Ch.Late.F	Forward primer to amplify late time point specific for patient A	5'- CGC GCTAGC AGT ACA TAT GTC ACC GGT GG-3'
NheI.AC.Early.F	Forward primer to amplify early time point specific for patient B	5'- CGC GCTAGC ACC ACA TAT ACC TCC GGT GGC-3'
NheI.AC.Late.F	Forward primer to amplify late time point specific for patient B	5'- CGC GCTAGC ACC ACA TAT ATC TCC GGT GGC-3'
**Mutants Internal Primers**		
Ch.HVR2.F	Forward primer to amplify HVR2 mutation (R466K) in patient A	5'- CCC ATC ACT TCC TTC AAG CAG GGA TGG-3'
Ch.HVR2.R	Reverse primer to amplify HVR2 mutation (R466K) in patient A	5'- CCA TCC CTG CTT GAA GGA AGT GAT GGG-3'
Ch.igVR.F	Forward primer to amplify igVR mutations (E575G/N577D/P578H) in patient A	5'-CCT TGT AAC ATC TAC GGG GGT GGG GGG GAT CAC GCC AAT GAT TCA GAC C-3'
Ch.igVR.R	Reverse primer to amplify igVR mutations (E575G/N577D/P578H) in patient A	5'- GGT CTG AAT CAT TGG CGT GAT CCC CCC CAC CCC CGT AGA TGT TAC AAG G-3'
AC.CD81-I.F	Forward primer to amplify CD81-I mutations (I414V/T416A) in patient B	5'-G CAG CTG GTC AAC GCC AAT GGC TCG TGG C-3'
AC.CD81-I.R	Reverse primer to amplify CD81-I mutations (I414V/T416A) in patient B	5'- G CCA CGA GCC ATT GGC GTT GAC CAG CTG C-3'
AC.CD81-II.F	Forward primer to amplify CD81-II mutations (A440G/R444H/K446R) in patient B	5'- GCT GGG TTG TTT TAT CAT TAT AGG TTC -3'
AC.CD81-II.R	Reverse primer to amplify CD81-II mutations (A440G/R444H/K446R) in patient B	5'- GAA CCT ATA ATG ATA AAA CAA CCC AGC -3'
AC.igVR.F	Forward primer to amplify igVR mutations (P574L/N576K) in patient B	5'- CCT TGC AAC ATC TAT GGG GAT AGG AGG GGT CTC AGT AAG AAC GG-3'
AC.igVR.R	Reverse primer to amplify igVR mutations (P574L/N576K) in patient B	5'- CC GTT CTT ACT GAG ACC CCT CCT ATC CCC ATA GAT GTT GCA AGG-3'
