# Supplementary material for: Longitudinal Sequence and Functional Evolution within Glycoprotein E2 in Hepatitis C Virus Genotype 3a Infection
Source: PLoS One. 2015 May 13;10(5):e0126397. doi: 10.1371/journal.pone.0126397 (PMC4430534; doi:10.1371/journal.pone.0126397)
Supplement: S4 Table — (PDF) [file pone.0126397.s007.pdf]

S4 Table. dN/dS analysis of sequences derived form this study.

Codon[a]	Region	dN/dS[b]	[illegible]	[illegible]
			[illegible]	MEME
Patient A (mean dN/dS = 0.74)				
233	E1	6.91	0.113	***0.004***
398	E2 (HVR1)	3.60	0.298	***0.004***
405	E2 (HVR1)	9.64	***0.018***	***0.017***
495	E2	7.11	0.111	***0.002***
501	E2	5.63	0.181	***0.024***
521	E2 (Epitope III)	10.80	***0.026***	***<0.001***
524	E2 (Epitope III)	6.00	0.132	***0.027***
Patient B (mean dN/dS = 0.76)				
260	E1	4.45	0.441	***0.015***
493	E2	5.94	0.296	***0.030***

[a]Codon positions are relative to the prototype strain H77c.

[b]Normalized dN/dS values determined by Single-Likelihood Ancestor Counting (SLAC). Evidence of positive selection is indicated by dN/dS>1, where dN and dS are the rates of non-synonymous and synonymous change, respectively.

[c]The level of signficance accepted in the SLAC and the Mixed Effects Model of Evolution (MEME) analyses were p-values<0.05. Those sites with any evidence of significant positive selection are highlighted with bolded and italicised font. Furthermore, sites with significance according to both methods are coloured red.
